# FZD7 Shapes Mitochondrial Function and Oxidative Stress in Hepatocellular Carcinoma: A Potential Prognostic Biomarker

**DOI:** 10.1002/biof.70156

**Published:** 2026-10-06

**Authors:** Peishu Huang, Fuqun Wei, Yiping Chen, Yuanmeng Yang

**Affiliations:** ^1^ Department of Radiology Shanghai Sixth People's Hospital Fujian Jinjiang China; ^2^ Department of Interventional Radiology First Affiliated Hospital of Fujian Medical University Fuzhou China; ^3^ Department of Interventional Radiology, National Regional Medical Center, Binhai Campus of the First Affiliated Hospital Fujian Medical University Fuzhou China

**Keywords:** FZD7, hepatocellular carcinoma, immunotherapy, mitochondrial metabolism, oxidative stress

## Abstract

Mitochondrial metabolism and oxidative stress (OS) play critical roles in the progression of hepatocellular carcinoma (HCC). However, reliable prognostic biomarkers for these processes remain unidentified. This study integrated bulk RNA sequencing and single‐cell RNA sequencing (scRNA‐seq) to identify OS&mitochondrial metabolism‐related genes (OMRGs). A prognostic model was constructed using LASSO regression, followed by external validation. Associations of the model with clinicopathological features, tumor microenvironment (TME), immune functions, therapeutic response, and drug sensitivity were systematically explored. Finally, the relationships between key OMRGs and mitochondrial metabolism as well as OS were validated. A risk score based on five key OMRGs (LPCAT1, IMPDH1, RAMP3, FZD7, and B4GALT5) was established. Low OMRS was significantly associated with improved overall survival and identified as an independent prognostic factor. Two subtypes (C1/C2) were characterized based on the OMRGs: the C1 subtype exhibited poorer survival and epithelial‐mesenchymal transition (EMT) enrichment, whereas the C2 subtype showed greater immune infiltration and sensitivity to immunotherapy. High OMRS was associated with higher predicted IC_50_ values to oxaliplatin and cisplatin, while low OMRS indicated favorable responses to TACE, sorafenib, and immunotherapy. FZD7 knockdown increased reactive oxygen species (ROS) levels and reduced mitochondrial membrane potential in HCC cells. FZD7, a key gene among OMRGs, represent promising prognostic biomarkers and therapeutic targets in HCC.

## Graphical abstract

OMRGs define distinct HCC subtypes with different survival outcomes, immune microenvironments, and predicted therapy responses. FZD7 is associated with prognosis and regulates mitochondrial function and oxidative stress, highlighting its potential as a therapeutic target.

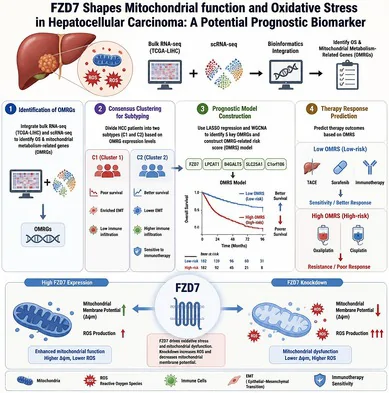

**Abbreviations**
DEGs: Differentially expressed genes
EMT: Epithelial‐mesenchymal transition
GDSC: Genomics of Drug Sensitivity in Cancer
GO: Gene Ontology
GSEA: Gene set enrichment analysis
GSVA: Gene set variation analysis
HCC: Hepatocellular carcinoma
KEGG: Kyoto Encyclopedia of Genes and Genomes
MnSOD: Mitochondrial superoxide dismutase
OMRGs: OS&mitochondrial metabolism‐related genes
OS: Oxidative stress
OS&Mito: OS&mitochondrial metabolism
qPCR: Quantitative PCR
ROC: Receiver operating characteristic
ROS: Reactive oxygen species
RS: Risk score
scRNA‐seq: Single‐cell RNA‐seq
TACE: Transarterial chemoembolization
TMB: Tumor mutation burden
TME: Tumor microenvironment

## 1 Introduction

Hepatocellular carcinoma (HCC), the predominant subtype of primary liver cancer, constitutes over 80% of cases globally and remains a malignancy of high morbidity and mortality, with approximately 830,000 deaths reported in 2020 [1, 2]. Current therapeutic strategies, including surgical resection and molecularly targeted therapies, face significant limitations due to frequent late‐stage diagnoses and intrinsic tumor heterogeneity, resulting in suboptimal clinical outcomes [3, 4]. Despite advancements in elucidating HCC's molecular mechanisms, the absence of reliable biomarkers for early detection and prognostic stratification persists, underscoring the imperative for innovative approaches.

Mounting evidence delineates the synergistic interplay between mitochondrial metabolism and OS imbalance in HCC pathogenesis [5, 6]. As the cellular energy hub, mitochondria govern not only ATP production via oxidative phosphorylation but also modulate reactive oxygen species (ROS) dynamics and apoptosis signaling. HCC tissues exhibit higher mitochondrial DNA mutation density compared to normal liver, directly impairing electron transport chain efficiency and elevating ROS generation [7]. This persistent oxidative stress (OS) exacerbates mitochondrial membrane potential depolarization, establishing a self‐perpetuating cycle of metabolic dysregulation [8]. Mechanistically, compromised cytochrome c release disrupts caspase‐mediated apoptosis, concurrent with a 50% downregulation of PINK1/Parkin‐dependent mitophagy, leading to pathological mitochondrial accumulation [9, 10]. Clinically, diminished mitochondrial superoxide dismutase (MnSOD) activity correlates with reduced overall survival [10], while elevated serum 8‐OHdG is associated with higher postoperative recurrence risk in HCC patients [11]. Notably, patients with > 30% reduction in mitochondrial complex IV activity demonstrate 58% lower response rates to sorafenib, highlighting the therapeutic implications of mitochondrial‐ROS axis perturbations [12]. Integrating mitochondrial metabolism biomarkers with markers of OS holds promise for enhancing prognostic accuracy and facilitating personalized therapeutic strategies for this challenging malignancy.

This study uses a multi‐dimensional bioinformatics approach to identify OS&mitochondrial metabolism‐related genes (OMRGs) linked to HCC patient survival. The main goal is to create and validate a prognostic model based on OMRGs to improve HCC patient survival and treatment efficacy, offering insights into mitochondrial metabolism and OS for personalized treatment strategies.

## 2 Materials and Methods

### 2.1 Data and Gene Sets Acquisition

The TCGA database provided bulk RNA‐seq data and clinical info for 343 HCC samples, while single‐cell RNA sequencing (scRNA‐seq) profiles (GSE149614) were analyzed using Seurat. Prognostic validation was done with GSE14520 (*n* = 221) and ICGC (*n* = 231), and additional GEO cohorts (GSE109211, *n* = 67; GSE104580, *n* = 147) assessed key OMRGs.

For the TCGA training dataset (RNA‐seq data), the RSEM normalized expression matrix was used, and expression values were transformed with log2(*x* + 1) to reduce skewness in the data. For the external validation datasets from the GEO and ICGC platforms (microarray data), already normalized and log2‐transformed expression matrices obtained from each dataset were further processed. We applied the normalizeBetweenArrays function in the R package limma to minimize inter‐sample variation.

For microarray datasets, probes were mapped to gene symbols using the platform‐specific annotation files. If multiple probes were mapped to the same gene, the probe with the highest expression value across the dataset was retained, based on the rationale that the probe with the strongest signal most accurately reflects gene expression. Probes that could not be mapped to any gene symbol were excluded from subsequent analyses to avoid introducing artifacts.

Four ICB‐treated cohorts were integrated into an immunotherapy dataset of 51 HCC samples via SVA to mitigate batch effects. Specifically, after obtaining the intersection of genes across the four immunotherapy cohorts, gene‐expression values were log2‐transformed using log2 (expression value + 1) and integrated into a single expression matrix. The ComBat function from the R package SVA was applied to correct batch effects originating from the four independent cohorts. Batch variables were defined according to cohort origin; the four cohorts were designated as Batch A, Batch B, Batch C and Batch D, with Batch A set as the reference batch. Treatment response was incorporated into the design matrix, in which patients were stratified as responders or non‐responders, so that biological differences associated with treatment response were preserved during batch‐effect correction. Parametric empirical Bayes estimation was utilized. The resulting batch‐corrected expression matrix was used for downstream analyses. Gene sets related to OS responses included 414 genes (Table S2) from 17 ROS pathways (Table S1) and 461 genes from 71 mitochondrial metabolic pathways in MitoCarta 3.0 (Table S3).

### 2.2 Acquisition of OMRGs From Bulk Transcriptomes by WGCNA

To discover novel OMRGs associated with overall OS and mitochondrial metabolism, gene set variation analysis (GSVA) was carried out. The GSVA analysis employed the intersection genes of OS‐related genes and those related to mitochondrial metabolism to calculate a GSVA OS&mitochondrial metabolism (OS&Mito) score, which was based on the “IOBR” package. Subsequently, WGCNA was implemented with the WGCNA R package. Genes expressed (> 0) in over 50% of samples and with expression > 10 in over 50% of samples were kept. The top 3500 genes with the highest median absolute deviation (MAD) were used for network building. Sample quality was assessed via hierarchical clustering and goodSamplesGenes; no outlier samples were excluded. Based on scale‐free topology criteria, a soft‐thresholding power of 10 was chosen. An unsigned co‐expression network was constructed using blockwiseModules with parameters: minModuleSize = 20, deepSplit = 4, reassignThreshold = 0, pamRespectsDendro = FALSE, mergeCutHeight = 0.25. Module eigengenes were computed and correlated with the OS&Mito phenotype. Modules with statistically significant correlations (*p* < 0.05) were retained for downstream analyses.

### 2.3 Acquisition of OMRGs by Comprehensive scRNA‐Seq Analysis

scRNA‐seq data (10 HCC samples) were processed using Seurat v5.0.1. Sequencing data were imported using the CreateSeuratObject function, and cells were filtered based on RNA counts ≥ 700, detected features ≥ 500, and mitochondrial gene ratio < 20%, leaving 71,742 cells for subsequent analyses. The data were normalized using NormalizeData, and the top 2000 highly variable genes were selected for PCA. The first 15 principal components identified by ElbowPlot were used for downstream analysis. Clustering was performed at a resolution of 0.3, yielding 24 clusters [13]. t‐SNE was used for dimensionality reduction and visualization. The “AUCell” package calculated cell activity scores based on the intersection genes of OS‐related genes and those related to mitochondrial metabolism. The AUCell_exploreThresholds function identified active cells, and TSNE colored cell clusters by AUC for OS&Mito response activity. The FindAllMarkers function identified DEGs between active and inactive subgroups.

### 2.4 Generation and Validation of the OS&Mito‐Related Prognostic Signature

The intersection of genes shared between TCGA‐LIHC and external validation datasets was identified and designated as the candidate gene set. The association between this candidate gene set and prognosis in TCGA‐LIHC was analyzed using univariable Cox regression to identify significant genes associated with HCC prognosis (*p* < 0.05), followed by intersection with differentially expressed genes (DEGs) from single‐cell data and Module genes correlated with OS&Mito scores. LASSO regression determined ideal OMRGs and their coefficients for the signature, dividing patients into high‐risk and low‐risk groups based on median risk score (RS). A random seed of 10,210 was set to ensure reproducibility. Ten‐fold cross‐validation was performed, and the *λ* value corresponding to the minimum cross‐validation partial likelihood deviance (“lambda.min”) was selected as the optimal lambda. Genes with non‐zero coefficients at the selected *λ* were retained for the final model.

The RS for individual samples in both TCGA‐LIHC and external validation datasets was computed using the formula:

$$\mathrm{RS}=\sum_{i=1}^{n}\mathrm{\beta i}\times {\mathrm{Exp}}_{i}$$

βi represents the model coefficient for the gene in the TCGA‐trained model, and ${\mathrm{Exp}}_{i}$ corresponds to the normalized and standardized expression value of the same gene in either the training or validation dataset. This approach ensured that the predictive performance of the prognostic model remained robust and consistent across platforms, despite potential differences in experimental designs or data formats.

Kaplan–Meier (KM) curves and receiver operating characteristic (ROC) curves were used for survival prediction and model validation, and a nomogram predicted 1‐year, 3‐year, and 5‐year survival probabilities for HCC patients.

### 2.5 Consensus Cluster Analysis

To subtype the HCC patients, we employed a consensus clustering algorithm based on the expression of lasso genes using ConsensusClusterPlus package. Finally, the maximum number of evaluated clusters was *k* = 2 based on the minimum PAC value in TCGA‐LIHC and the validation dataset (Table S4).

### 2.6 Enrichment Analysis

Gene Ontology (GO) and Kyoto Encyclopedia of Genes and Genomes (KEGG) analyses were performed on the two subtype groups of patients with HCC using the “clusterProfiler” R package, “circlize” R package visualizes the GO and KEGG results. In addition, gene set enrichment analysis (GSEA) of KEGG terms using clusterProfiler was performed.

### 2.7 Analysis of the Immune Infiltration Microenvironment and Effective Immunotherapy Response in Different Subtypes

CIBERSORT, EPIC, ESTIMATE, MCP counter, QUANTISEQ, and TIMER algorithm, utilizing the “IOBR” R package, calculated the proportion of infiltrating immune cells in different subtype groups of the TCGA‐LIHC datasets. In addition, the Tumor Immune Dysfunction and Exclusion (TIDE) algorithm was performed to assess ICB therapy responses and evaluate the ability to serve as a neoantigen (http://tide.dfci.harvard.edu).

### 2.8 Evaluation of Therapeutic Benefit and Drug Sensitivity

The LASSO‐derived RS formula was applied to the integrated immunotherapy dataset and transarterial chemoembolization (TACE) and sorafenib datasets, predicting drug sensitivity using the “pRRophetic” R package, while Spearman's correlation assessed the relationship between half‐maximal inhibitory concentration (IC50) and gene expression, with a box plot showing IC50 distribution across subtypes. Detailed information of the Treatment Cohorts is shown in Table S5.

### 2.9 Gene Mutation Analysis

The “maftools” R package created waterfall plots for genes with high somatic mutation frequencies. We calculated the tumor mutation burden (TMB) score for each HCC patient and explored the differences between different subtype groups.

### 2.10 Cell Culture and Transfection

HuH‐7 cells were sourced from Wuhan Procell (Cat# CL‐0103, Wuhan, China) and cultured in high‐glucose DMEM (Procell, Cat# PM150210) with 20% FBS, penicillin, and streptomycin (all purchased from Beijing Solarbio Science & Technology Co. Ltd., Cat# P1400), maintained at 37°C with 5% CO_2_, and passaged at 90% confluency using trypsin–EDTA (Procell, Cat# PB180226).

HuH‐7 cells were seeded in 48‐well plates at 2 × 10^4^ cells per well, and FZD7 or control siRNA (GenePharma Co. Ltd., Shanghai, China) was mixed with Lipofectamine 3000 (Invitrogen, MA, USA) in serum‐free medium, incubated for 20 min, then added to complete medium with 10% FBS. Cells were incubated for 38 h post‐transfection. The siRNA sequences used were as follows:

- FZD7 siRNA‐1: Sense: 5′‐GAGCUUGAAUUCUCAATT‐3′, Antisense: 5′‐UUGAGAGAAAUUCAAGCUCTT‐3′;
- FZD7 siRNA‐2: Sense: 5′‐AUCGAACGCAGGAAGAUCATT‐3′, Antisense: 5′‐UGAUCUUCCUGCGUUCGAUTT‐3′;
- siRNA (si‐control): Sense: 5′‐UUCUCCGAACGUGUCACGUTT‐3′, Antisense: 5′‐ACGUGACACGUUCGGAGAATT‐3′.

Transfection efficiency was verified by quantitative PCR (qPCR). The experiment was divided into three groups: untreated control (WT), negative control siRNA‐transfected group (NC), and FZD7 siRNA‐transfected group (si‐FZD7), with three biological replicates per group (*n* = 3).

### 2.11 ROS Detection

Intracellular ROS levels were measured using a ROS Assay Kit (Beyotime Biotechnology, Cat# S0033S, Shanghai, China), following the manufacturer's protocol. Cells were harvested, washed with PBS, and resuspended in diluted DCFH‐DA solution, then incubated at 37°C. After washing, ROS levels were analyzed by flow cytometry.

### 2.12 Mitochondrial Membrane Potential (MMP) Detection

After treatment, supernatant from six‐well plates was collected, cells washed with PBS, digested with trypsin, and combined with supernatant. The suspension was centrifuged, supernatant discarded, and cells washed twice with PBS before incubation with JC‐1 solution (Solarbio, Cat# M8650, Beijing, China) at 37°C for 20 min. Post‐incubation, cells were centrifuged, staining solution removed, washed with JC‐1 buffer, and resuspended in buffer. Mitochondrial membrane potential was analyzed by flow cytometry using FITC‐A and PE‐A channels.

### 2.13 Statistical Analyses

Statistical analyses were conducted using R (version 4.4.1), employing Cox regression for prognostic gene screening, Kaplan–Meier for survival analysis, Wilcoxon rank‐sum for two‐group comparisons, and Kruskal‐Wallis for multiple groups. Significance levels were indicated as NS, *p* > 0.05; *, *p* ≤ 0.05; **, *p* ≤ 0.01; ***, *p* ≤ 0.001; and ****, *p* ≤ 0.0001.

## 3 Results

### 3.1 WGCNA Screened Module Genes Associated With OMRGs in TCGA‐LIHC

Our study identifies novel prognostic targets for HCC using 37 intersect genes linked to OS and mitochondrial metabolism (Figure 1a). GSVA assigned these genes to the TCGA‐LIHC cohort, followed by WGCNA to find OS&Mito‐related modules with an optimal soft‐threshold power of 10. The dendrogram demonstrated that the pink, purple, and blue modules showed the strongest correlations with OS&Mito scores (Figures 1b–d).

**FIGURE 1:**
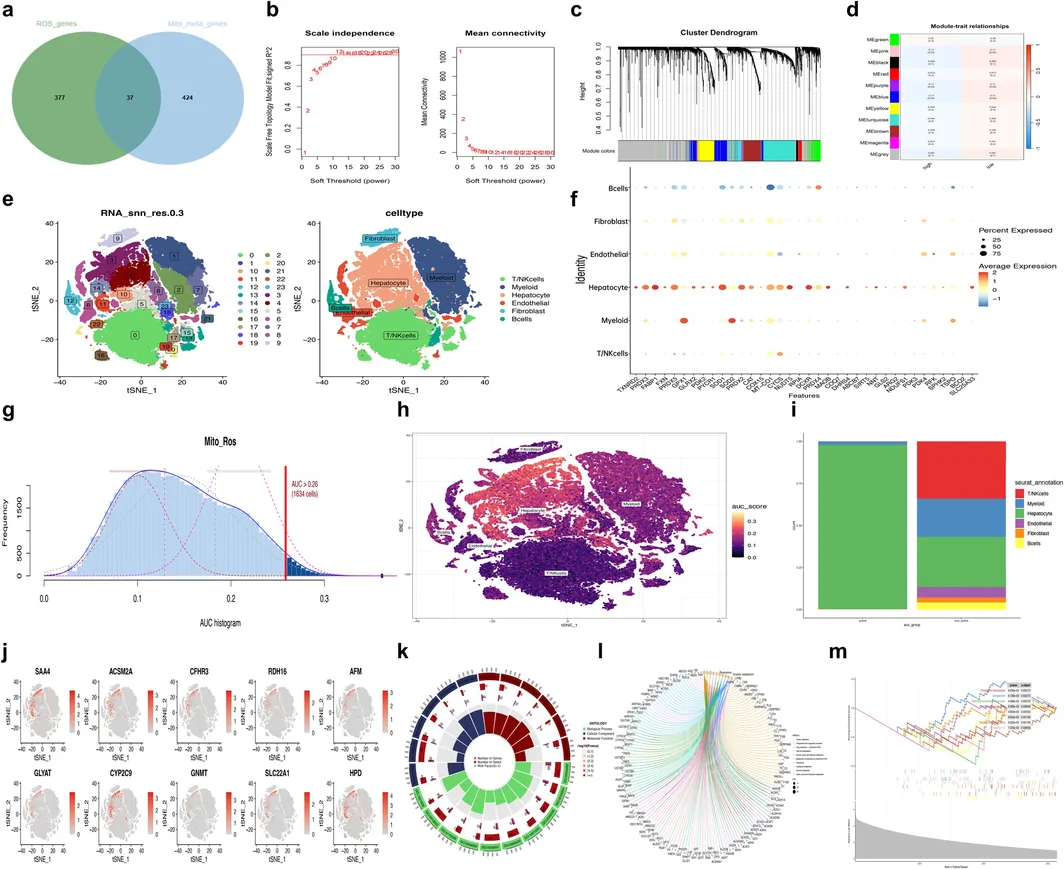
Identification and functional characterization of OMRGs. (a) Venn diagram showing the overlap between ROS‐related genes (green) and mitochondrial metabolism‐related genes (blue). (b) Scale‐free topology analysis for network construction. (c) Gene clustering dendrogram based on hierarchical clustering. (d) Heatmap illustrating the correlation between gene modules and the OS&Mito score. (e) t‐SNE plots displaying 24 clusters in the GSE149614 dataset (left) and their annotation into six distinct cell types (right). (f) Bubble plot showing the expression patterns of OMRGs across the six cell types. (g) Histogram of AUC scores for the OS&Mito gene signature across all cells; the vertical line indicates the activity classification cutoff (AUC > 0.26). (h) t‐SNE map colored by AUC scores, representing the expression intensity of the OS&Mito signature across different cell populations. (i) Bar plot showing the proportion of each cell type in high‐ and low‐AUC groups. (j) t‐SNE plots illustrating the expression of the top 10 marker genes in the high‐AUC group. (k–m) Functional enrichment analyses of DEGs between high‐ and low‐AUC groups, including GO, KEGG, and GSEA.

### 3.2 Acquisition of OMRGs by Comprehensive scRNA‐Seq Analysis

We processed the GSE149614 scRNA‐seq dataset with Seurat, identifying 24 clusters annotated into six cell types (Figure 1e), including myeloid cells and HCC cells. OMRGs expression was visualized in a dot plot, showing high expression in HCC cells (Figure 1f). The AUCell package calculated cell activity scores, identifying active cells with an AUC activity threshold of 0.26 (Figure 1g). t‐SNE plots showed HCC cells mainly in the high‐score group (Figures 1h,i). The top 10 genes in the active group were displayed (Figure 1j), and DEGs between active and inactive subgroups were identified, followed by GO, KEGG, and GSEA analyses. GO analysis revealed enrichment in metabolic pathways (Figure 1k), while KEGG identified pathways like Carbon metabolism and Drug metabolism (Figure 1l). GSEA indicated suppression of pathways such as Oxidative phosphorylation and Apoptosis (Figure 1m).

### 3.3 Development of the OS&Mito‐Related Signature

We identified 6344 prognostic genes in the TCGA‐LIHC cohort via Cox regression, filtered 13,670 candidate genes from single‐cell analysis, and intersected them with WGCNA results to find 185 promising OMRGs (Figure 2a). Five OMRGs were selected using LASSO regression to construct the signature (Figure 2b,c), with their expression levels generating the OMRS for each sample (Figure 2d). The risk score was calculated as follows: RS = 0.13256940 × LPCAT1 + 0.04352273 × IMPDH1–0.13240468 × RAMP3 + 0.01815332 × FZD7 + 0.01170158 × B4GALT5. In the LIHC cohort, the low‐OMRS group showed reduced macrovascular invasion, lower AFP levels, and earlier tumor stages (Figures 2e–g). OMRS values were calculated for TCGA‐LIHC samples, grouping them based on median risk score; the low‐OMRS group showed significantly longer overall survival, validated in GSE14520 and ICGC cohorts (Figure 2h), with time‐dependent ROC curves indicating strong prognostic accuracy (Figure 2i). A multivariate Cox regression analysis confirmed that OMRS is an independent prognostic factor for LIHC (Figure 2j), leading to the creation of a predictive nomogram that includes age, sex, AFP, CTP, tumor stage, and OMRS for estimating survival probabilities, with calibration analyses showing high accuracy of the model (Figures 2k,l).

**FIGURE 2:**
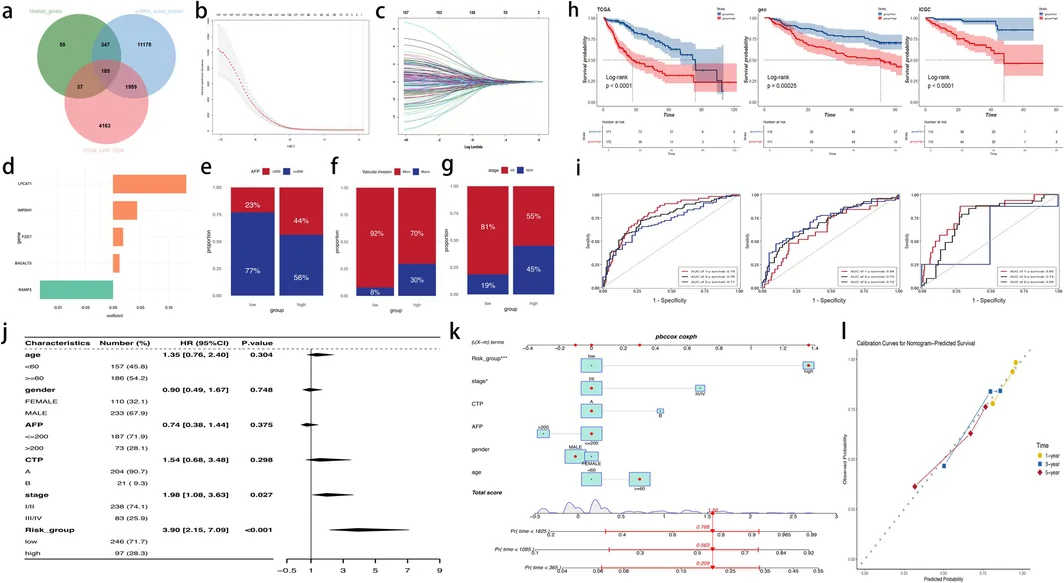
Construction and Validation of the OMRS Prognostic Model. (a) Venn diagram illustrating the overlap among OS&Mito‐active genes, OS&Mito‐related module genes, and prognostic genes derived from the TCGA dataset. (b and c) LASSO regression analysis for the identification of signature genes. (d) Coefficient values of each gene in the OMRS model. (e–g) Histograms and box plots showing that the high‐OMRS group is associated with elevated AFP levels, macrovascular invasion, and advanced tumor stage. (h and i) Kaplan–Meier survival curves comparing high‐ and low‐OMRS groups, along with time‐dependent ROC curves evaluating the predictive performance of OMRS in the TCGA‐LIHC, GSE14520, and ICGC cohorts. (j) Forest plot demonstrating that OMRS remains an independent prognostic factor after multivariate adjustment for age, sex, AFP levels, liver function, and tumor stage in the TCGA‐LIHC cohort. (k) Nomogram for individualized survival prediction. (l) Calibration curves showing the agreement between predicted and observed survival probabilities at 1‐, 3‐, and 5‐year time points.

### 3.4 Identification of OS&Mito Subtypes Based on OMRGs

Given the aforementioned results indicating that OMRGs are associated with the clinicopathological features and prognosis of HCC, we performed consensus clustering analysis based on OMRGs to classify patients in the TCGA‐LIHC cohort into two clusters: C1 and C2 (Figure 3a). DEGs between the two clusters were identified using the “limma” package (Figure 3b). Kaplan–Meier survival analysis was then conducted to evaluate the association between the clusters and the overall survival of HCC patients. The results revealed that patients in different clusters exhibited significantly distinct overall survival outcomes, with the C1 cluster showing a markedly worse overall survival (Figure 3c). Functional enrichment analyses, including GO, KEGG, and GSEA, were performed on the DEGs between the two clusters (Figures 3d–f). The analyses revealed pathways associated with metastasis and metabolic processes, and epithelial‐mesenchymal transition (EMT) was significantly inhibited in cluster C2.

**FIGURE 3:**
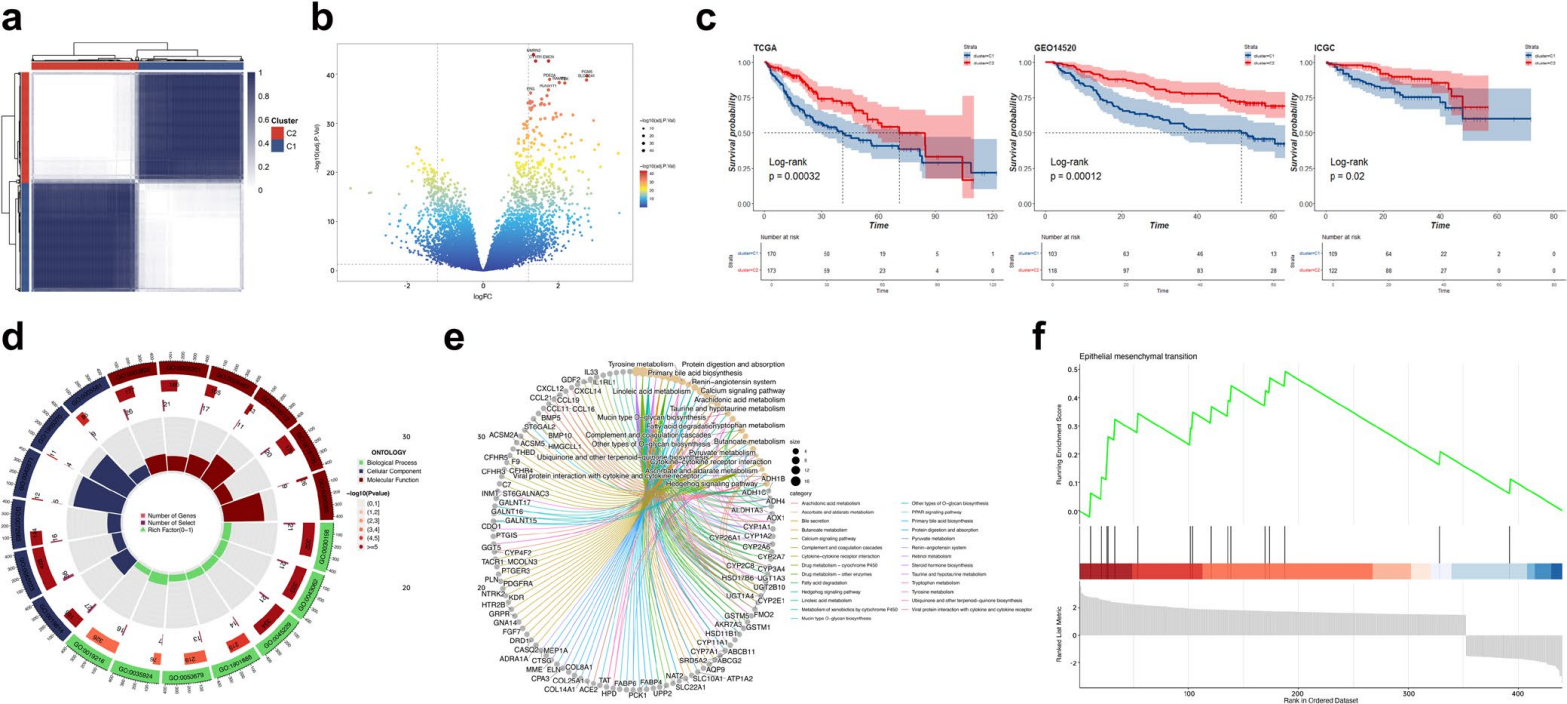
Molecular Subtyping Based on OMRS and Functional Characterization. (a) Consensus clustering analysis of LIHC samples from the TCGA‐LIHC cohort. (b) Volcano plot showing differentially expressed genes (DEGs) between the two identified subtypes in TCGA‐LIHC. (c) Kaplan–Meier survival curves comparing overall survival between the two subtypes in the TCGA‐LIHC, GSE14520, and ICGC cohorts. (d–f) Functional enrichment analyses of DEGs between the two subtypes in the TCGA‐LIHC cohort, including GO, KEGG, and GSEA.

### 3.5 Biological Differences Between Subtypes

Significant differences in immune checkpoint gene expression levels and correlations were found across subtypes in three datasets (Figure S1a–f). KEGG pathway analysis showed a consistent positive association between oxidative phosphorylation and identified clusters (Figure S1g–i). This validates OMRGs' ability to distinguish OS&Mito‐related subtypes. We used the IOBR package to analyze immune infiltration in subtypes, finding C2 had higher CD8+ T cells and macrophages than C1 (Figure 4a). The TIDE algorithm showed C1 had a higher TIDE score and lower MSI than C2, suggesting C2 patients may benefit more from immunotherapy (Figures 4c–e). Mutation analysis revealed 39% of C1 patients had TP53 mutations, while 34% of C2 patients had CTNNB1 mutations (Figure 4b).

**FIGURE 4:**
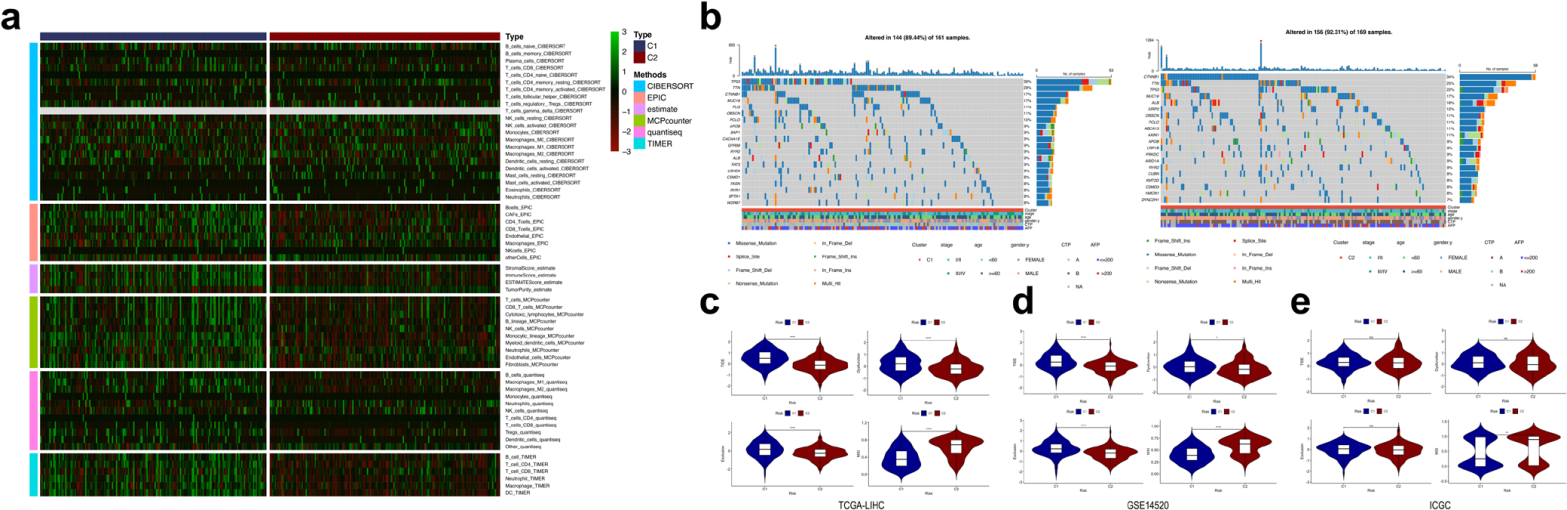
Immune Landscape and Tumor Microenvironment Characteristics Across Subtypes. (a) Heatmap illustrating immune cell infiltration patterns across different subtypes, as estimated by multiple algorithms in the TCGA‐LIHC cohort. (b) TMB across different subtypes. (c–e) Comparison of tumor immune microenvironment (TIME) scores among subtypes across three independent datasets.

### 3.6 Varied Treatment Responses Between Subtypes

To determine whether the model can predict responses to immunotherapy, TACE, and sorafenib, the datasets were divided into high‐risk and low‐risk groups based on RS cutoff to assess model predictions for immunotherapy, TACE, and sorafenib. A cumulative distribution plot showed that low‐risk patients responded better to treatments than high‐risk patients (Figures 5b,d,f), and responders had lower risk scores than non‐responders in the TACE group (Figures 5a,c,e). IC50 values of 138 drugs from the Genomics of Drug Sensitivity in Cancer (GDSC) database were predicted from TCGA‐LIHC data (Table S6), showing significant differences between high‐risk and low‐risk groups (Figure 5g). The RS predicted IC50 values for Dactinomycin, Vinorelbine, Oxaliplatin, Cyclophosphamide, Epirubicin, and Cisplatin, with LPCAT1 and B4GALT5 positively correlating with Oxaliplatin and negative correlations for Vinorelbine, Epirubicin, and Cisplatin (Figure 5h).

**FIGURE 5:**
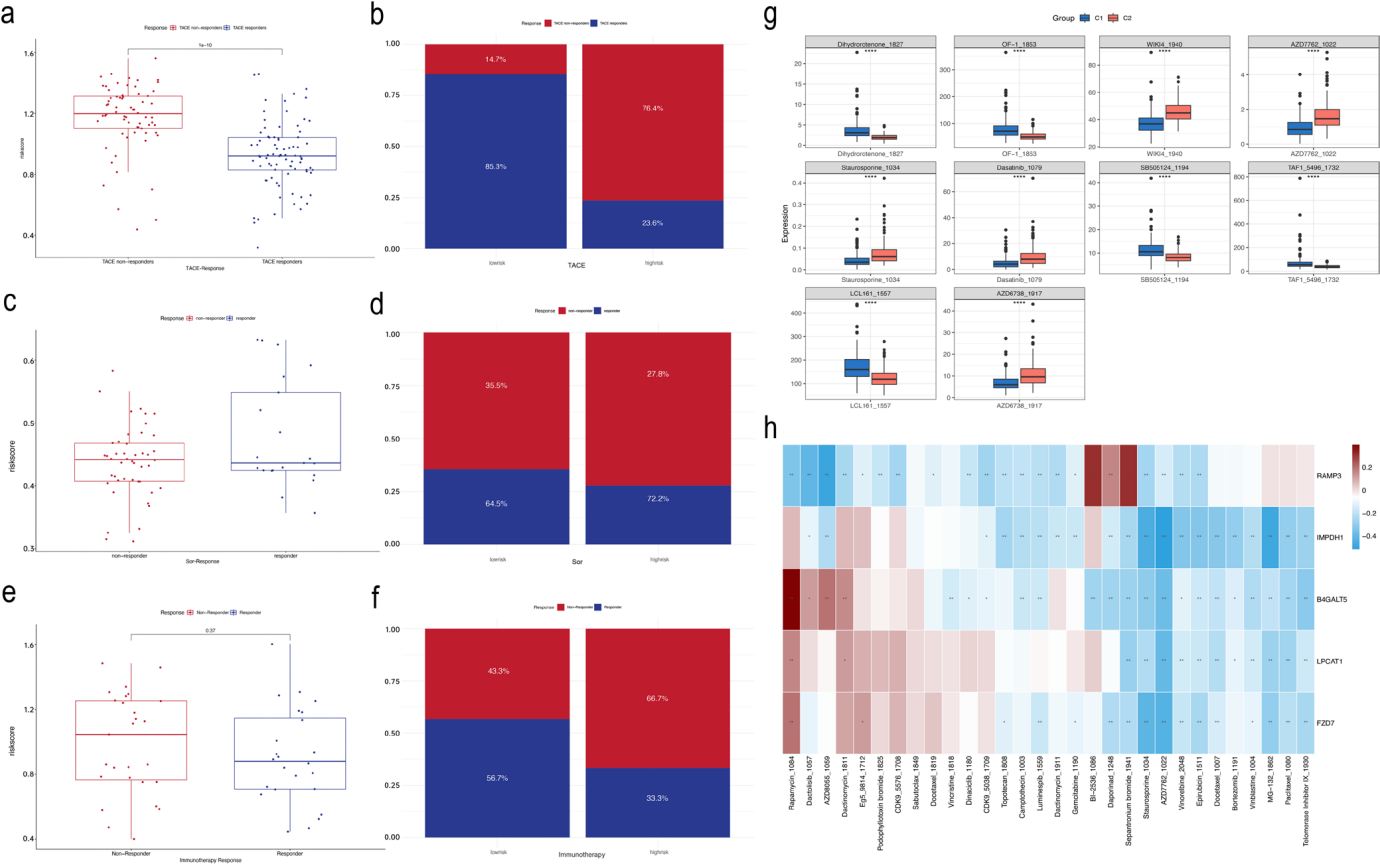
Therapeutic Response and Drug Sensitivity Associated with OMRS. (a–f) Scatter plots and box plots showing differences in OMRS across various therapeutic responses, along with the proportions of treatment responses between high‐ and low‐OMRS groups under different therapies. (g) Predicted IC50 values of the top 10 most significantly differential drugs across subtypes in the TCGA‐LIHC cohort. (h) Heatmap illustrating the correlations between OMRGs and drug sensitivity.

### 3.7 Effect of FZD7 Knockdown on Intracellular ROS Levels

The impact of FZD7 knockdown on ROS levels in HuH‐7 cells was assessed using the DCFH‐DA probe and quantified by flow cytometry, with three groups (WT, NC, si‐FZD7) and three biological replicates each; FZD7 knockdown efficiency was validated by qPCR (Figure 6a). In the WT group, the proportion of ROS‐positive cells was 18.02%, and the NC group showed a comparable level of 16.58%, indicating low basal ROS levels in HuH‐7 cells. No obvious change was observed in the NC group, suggesting that negative‐control siRNA exerted minimal effects on cellular ROS. By contrast, the si‐FZD7 group exhibited a markedly elevated fraction of ROS‐positive cells (30.03%, Figure 6b), with a statistically significant difference relative to the NC group (*t* = 2.87, *p* < 0.05; Figure 6c). These data indicate that FZD7 knockdown promotes ROS accumulation in HuH‐7 cells.

**FIGURE 6:**
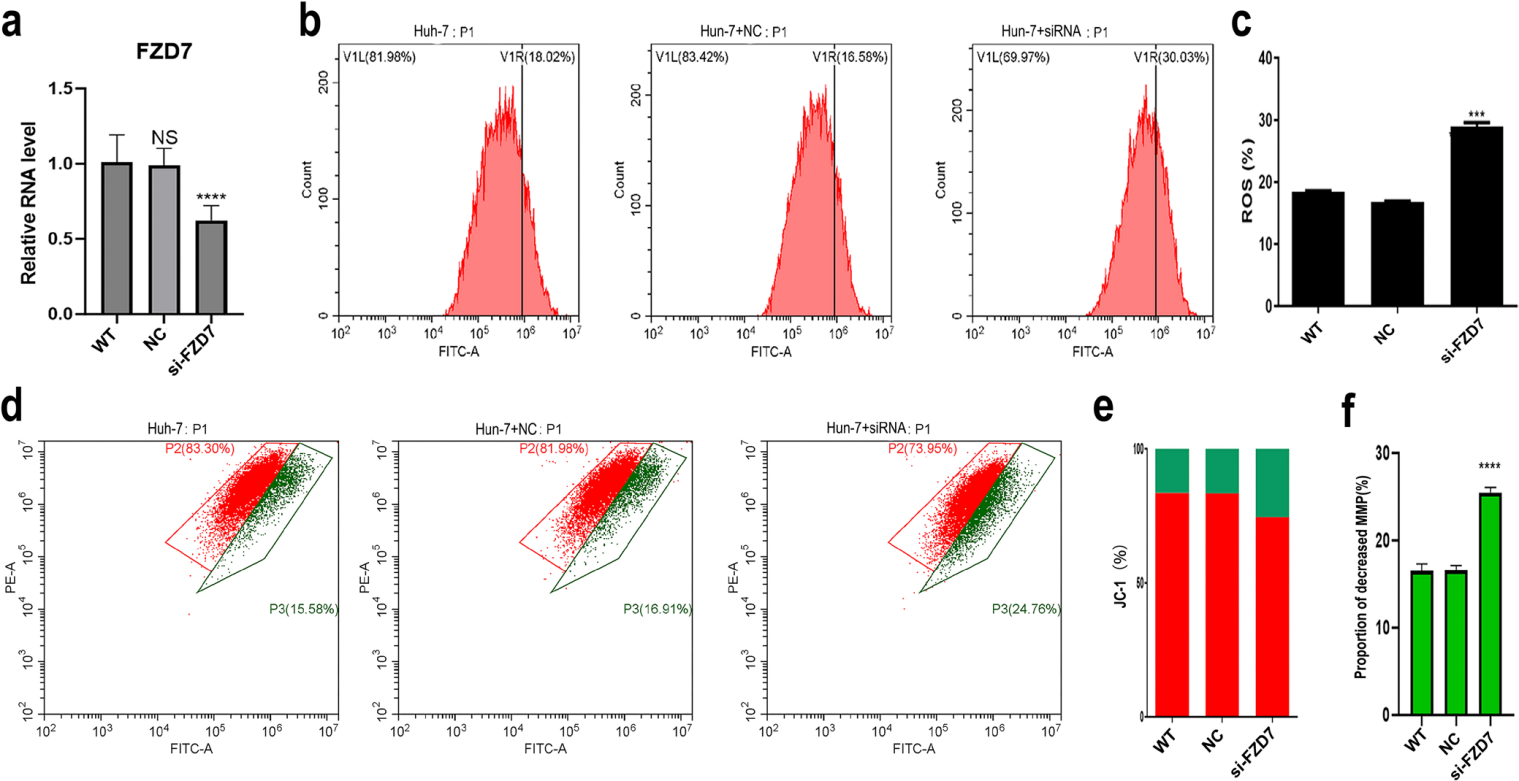
Functional Validation of FZD7 in ROS Production and Mitochondrial Dysfunction. (a) mRNA expression levels of FZD7 in WT, NC, and si‐FZD7 groups; experiments were performed in triplicate. (b) ROS levels measured by flow cytometry in different groups. (c) Quantitative comparison of ROS levels among groups. (d) Mitochondrial membrane potential (MMP) assessed by flow cytometry using JC‐1 staining; red fluorescence indicates JC‐1 aggregates in healthy mitochondria, whereas green fluorescence indicates JC‐1 monomers in depolarized mitochondria. (e) Quantification of JC‐1 staining results presented as a bar plot. (f) Comparison of the proportion of cells with decreased MMP among different groups.

### 3.8 Effect of FZD7 Knockdown on MMP

JC‐1 staining assessed the impact of FZD7 knockdown on mitochondrial function by measuring changes in MMP, with high MMP cells emitting red fluorescence and low MMP cells emitting green fluorescence. In the WT group, 82.70% of cells were red‐fluorescent, indicating healthy mitochondria, with no significant differences between NC and WT groups (Figure 6d). However, in the si‐FZD7 group, red‐fluorescent cells decreased to 73.69%, while green‐fluorescent cells increased to 24.78%, showing significant impairment in mitochondrial function due to FZD7 knockdown (Figures 6e,f).

## 4 Discussion

HCC cells frequently undergo metabolic reprogramming during tumor progression, particularly through significant alterations in mitochondrial metabolic pathways [14, 15]. For instance, to meet the energy demands and biosynthetic precursor requirements of rapid proliferation, HCC cells often maintain survival by enhancing the activity of metabolic pathways such as fatty acid oxidation [16]. When mitochondrial metabolism becomes dysregulated—manifesting as electron transport chain dysfunction or diminished membrane potential—this leads to increased generation of ROS [17]. ROS not only serves as a metabolic byproduct but also plays pivotal roles in cellular signaling and metabolic regulation [18]. Moderate ROS levels can promote cell proliferation, whereas excessive accumulation may damage mitochondrial DNA, proteins, and lipids, compromising mitochondrial structure/function and ultimately exacerbating cellular stress or inducing apoptosis [19].

In this study, we identified a set of genes closely associated with OS and mitochondrial metabolism through integrative analysis. Single‐cell transcriptomic profiling revealed that these OMRGs were highly expressed in HCC cells, particularly within HCC cells. AUCell analysis further validated that these genes served as markers of highly active cancer cells, showing significant enrichment in metabolic pathways such as fatty acid metabolism and organic acid catabolism. Functional enrichment analyses, including GO, KEGG, and GSEA, consistently demonstrated that OS&Mito‐high cell subsets exhibited activated metabolic pathways, while stress‐related pathways such as apoptosis and hypoxia responses were significantly suppressed. These findings suggest that OS&Mito‐high cells possess enhanced survival capacity and adaptive mechanisms, potentially representing a more aggressive and resilient tumor subpopulation.

Based on the integration of multiple transcriptomic datasets, we constructed an OMRS model comprising five key OMRGs and systematically evaluated its prognostic value. The results demonstrated that patients with lower OMRS scores exhibited significantly prolonged overall survival, accompanied by favorable clinical characteristics, including lower AFP levels, earlier tumor stages, and a reduced incidence of macrovascular invasion. Multivariate Cox regression analysis further confirmed that the OMRS score served as an independent prognostic factor, maintaining statistical significance after adjusting for clinical variables such as age, gender, AFP levels, and TNM stage. Additionally, a nomogram incorporating the OMRS score was developed to quantitatively predict 1‐, 3‐, and 5‐year survival probabilities. The calibration curves showed excellent concordance between the predicted and observed outcomes, highlighting the robust clinical applicability of the model.

Furthermore, consensus clustering analysis stratified patients in the TCGA‐LIHC cohort into two molecular subtypes, designated C1 and C2. Patients classified under the C1 subtype exhibited significantly worse prognoses. Functional enrichment analysis revealed that the C1 subtype was associated with activation of tumor‐promoting processes such as “regulation of epithelial cell proliferation” and “cell junction assembly,” suggesting enhanced potential for tumor invasion and angiogenesis. In contrast, the C2 subtype showed suppressed EMT signaling and enhanced metabolic activity, indicating a more stable cellular phenotype with lower metastatic potential.

Immune microenvironment profiling also revealed substantial differences between the two subtypes. The C2 subtype was characterized by significantly higher infiltration of CD8^+^T cells, dendritic cells, and M1‐type macrophages, along with elevated ESTIMATE scores, suggesting a more immunologically active tumor microenvironment (TME). It is well established that the presence of CD8^+^ T cells is a hallmark of an immunologically “hot” tumor [20, 21], often associated with better responsiveness to immunotherapy [22, 23]. The enrichment of M1 macrophages further supports a pro‐inflammatory and anti‐tumor immune microenvironment in the C2 subtype [24, 25]. In contrast, the C1 subtype exhibited significantly higher TIDE scores and lower MSI scores, reflecting an enhanced immunosuppressive state and reduced responsiveness to immunotherapy. Elevated TIDE scores are typically indicative of T cell dysfunction or immune exclusion, while lower MSI scores suggest insufficient antigen load, both of which contribute to diminished efficacy of immune checkpoint inhibitors [26]. Immune checkpoints are inhibitory signaling pathways within the immune system that are often exploited by tumor cells to evade immune surveillance and suppress anti‐tumor responses [27]. Moreover, differential expression of immune checkpoint‐related genes, such as LAG3, CD28, and HLA‐DRB1, between the two subtypes further elucidates the molecular mechanisms underlying tumor immune evasion. These findings provide a theoretical basis for the development of precise and individualized immunotherapeutic strategies.

Therapeutic response analysis further demonstrated that patients with lower OMRS scores exhibited better responses to immunotherapy, TACE, and the targeted agent sorafenib, underscoring the potential clinical utility of the risk model in guiding treatment decisions. Chemosensitivity predictions revealed that patients in the high‐risk group had significantly higher IC50 values for commonly used chemotherapeutic agents such as oxaliplatin, epirubicin, and cisplatin, suggesting a lower predicted sensitivity. Moreover, core model genes—particularly LPCAT1 and B4GALT5—were strongly correlated with drug sensitivity, providing important insights for optimizing future chemotherapy combination strategies.

In vitro functional assays were conducted to further explore the regulatory role of FZD7 in redox homeostasis and mitochondrial function in HCC cells. Our results showed that FZD7 knockdown significantly increased intracellular ROS levels in HuH‐7 cells, suggesting its critical role in maintaining the antioxidant capacity of tumor cells. Moreover, JC‐1 staining demonstrated a marked decrease in mitochondrial membrane potential upon FZD7 silencing, indicating mitochondrial dysfunction and highlighting its importance in preserving mitochondrial integrity. Integrated with the OMRS risk model analysis, we found that FZD7 expression was significantly elevated in high‐risk patients, implicating it as a potential upstream regulator of metabolic reprogramming and OS responses in HCC. Previous studies have identified FZD7 as a key receptor of the Wnt/β‐catenin signaling pathway, where its overexpression is associated with enhanced tumor stemness, metastatic potential, and chemoresistance across various solid tumors [28, 29, 30]. In HCC specifically, FZD7 has been shown to promote cancer stem cell properties and survival, partly through modulating the expression of metabolism‐related genes downstream of Wnt signaling [31]. Thus, silencing FZD7 not only disrupts the cellular adaptation to OS but may also impair mitochondrial metabolic pathways, thereby reducing tumor cell viability and invasiveness. These findings suggest that FZD7 may serve as a promising therapeutic target. Its inhibition could provide a novel strategy for metabolic‐targeted therapies or combination immunotherapies in the treatment of HCC.

Despite the consistent findings obtained from multiple transcriptomic datasets and in vitro functional experiments, this study has several limitations. First, the primary data were derived from public databases, which may introduce potential biases related to sample heterogeneity and sequencing platform variability. Second, the OMRS model has not yet been prospectively validated in large‐scale, independent clinical cohorts, limiting its immediate clinical applicability. Moreover, the regulatory mechanisms linking FZD7 to the OS&Mito network remain incompletely understood. Future studies integrating CRISPR‐based functional screens, metabolomics, and other multi‐omics approaches are warranted to elucidate the precise role of FZD7 in OS and mitochondrial metabolism in HCC.

## 5 Conclusions

This study developed a prognostic model using OMRS, highlighting differences in immune microenvironment and treatment response among subtypes. The model shows strong predictive ability and supports personalized treatment, with FZD7 providing insights into HCC regulation. Future research should validate these findings and explore their clinical applications for precision medicine in HCC.

## Author Contributions

Conceptualization, formal analysis, writing – review and editing, supervision, and project administration: Y.Y. and Y.C. Methodology, software, data curation, and writing – original draft preparation: F.W. Validation, investigation, resources, and writing – original draft preparation: P.H. All authors have read and agreed to the published version of the manuscript.

## Funding

This study was funded by the “Medical Endless Exploration of Disease Diagnosis and Treatment” Project of Fujian Shangmeng Public Welfare Foundation.

## Ethics Statement

The authors have nothing to report.

## Consent

The authors have nothing to report.

## Conflicts of Interest

The authors declare no conflicts of interest.

## Supporting information


**Figure S1:** Immune Checkpoint Profiles and Pathway Correlations Across Subtypes. (a–c) Heatmaps illustrating the expression levels of immune checkpoint genes across different subtypes in three independent datasets. (d–f) Correlation analyses of immune checkpoint gene expression within each subtype across the corresponding datasets. (g–i) Correlation analyses between subtypes and KEGG pathways across the three independent datasets.

**Table S1:** Gene sets related to the OS response in 17 ROS pathways.

**Table S2:** 414 OS‐related genes.

**Table S3:** Genes related to mitochondrial metabolic pathways.

**Table S4:** Consensus cluster in TCGA‐LIHC, GSE14520 and ICGC dataset.

**Table S5:** Treatment Cohort Details.

**Table S6:** Prediction of IC50 values of 138 drugs in the GDSC database based on the TCGA‐LIHC expression profile.

## Data Availability

The datasets analyzed for this study can be found in the TCGA database (http://www. cancer.gov/tcga), the Gene Expression Omnibus (https://www.ncbi.nlm.nih.gov/geo), and the International Cancer Genome Consortium (https://dcc.icgc.org/releases/current/Projects).
