# Supplementary material for: FZD7 Shapes Mitochondrial Function and Oxidative Stress in Hepatocellular Carcinoma: A Potential Prognostic Biomarker
Source: Biofactors. 2026 Oct 6;52(5):e70156. doi: 10.1002/biof.70156 (PMC13643192; doi:10.1002/biof.70156)
Supplement: Supplementary file 1 [file BIOF-52-0-s002.docx]

**TableS5:** Treatment Cohort Details

| **GEO number** | **Platforms** | **Case** | **Treatment** |
| --- | --- | --- | --- |
| GSE279750 | GPL20301 | 10 | anti-PD-L1 therapy |
| GSE215011 | GPL11154 | 10 | Nivolumab |
| GSE202069 | GPL24676 | 66 | anti-PD-1 therapy |
| GSE181946 | GPL24676 | 17 | anti-PD-1 therapy |
| GSE109211 | GPL13938 | 140 | sorafenib |
| GSE104580 | GPL570 | 147 | Transarterial chemoembolization |
